# Effectiveness of proactive telephone counselling for smoking cessation in parents: Study protocol of a randomized controlled trial

**DOI:** 10.1186/1471-2458-11-732

**Published:** 2011-09-26

**Authors:** Kathrin Schuck, Roy Otten, Marloes Kleinjan, Jonathan B Bricker, Rutger CME Engels

**Affiliations:** 1 Behavioural Science Institute, Radboud University Nijmegen, Montessorilaan 3, P.O. Box 9104, 6500 HE Nijmegen, The Netherlands; 2 Fred Hutchinson Cancer Research Center, 1100 Fairview Avenue, P.O. Box 19024, Seattle, WA 98109, USA; 3 University of Washington, Department of Psychology, Box 351525, Seattle, WA 98195, USA

## Abstract

**Background:**

Smoking is the world's fourth most common risk factor for disease, the leading preventable cause of death, and it is associated with tremendous social costs. In the Netherlands, the smoking prevalence rate is high. A total of 27.7% of the population over age 15 years smokes. In addition to the direct advantages of smoking cessation for the smoker, parents who quit smoking may also decrease their children's risk of smoking initiation.

**Methods/Design:**

A randomized controlled trial will be conducted to evaluate the effectiveness of proactive telephone counselling to increase smoking cessation rates among smoking parents. A total of 512 smoking parents will be proactively recruited through their children's primary schools and randomly assigned to either proactive telephone counselling or a control condition. Proactive telephone counselling will consist of up to seven counsellor-initiated telephone calls (based on cognitive-behavioural skill building and Motivational Interviewing), distributed over a period of three months. Three supplementary brochures will also be provided. In the control condition, parents will receive a standard brochure to aid smoking cessation. Assessments will take place at baseline, three months after start of the intervention (post-measurement), and twelve months after start of the intervention (follow-up measurement). Primary outcome measures will include sustained abstinence between post-measurement and follow-up measurement and 7-day point prevalence abstinence and 24-hours point prevalence abstinence at both post- and follow-up measurement. Several secondary outcome measures will also be included (e.g., smoking intensity, smoking policies at home). In addition, we will evaluate smoking-related cognitions (e.g., attitudes towards smoking, social norms, self-efficacy, intention to smoke) in 9-12 year old children of smoking parents.

**Discussion:**

This study protocol describes the design of a randomized controlled trial to evaluate the effectiveness of proactive telephone counselling in smoking cessation. It is expected that, in the telephone counseling condition, parental smoking cessation rates will be higher and children's cognitions will be less favorable about smoking compared to the control condition.

**Trial registration:**

The protocol for this study is registered with the Netherlands Trial Register NTR2707.

## Background

Cigarette smoking continues to be a serious problem with detrimental health consequences for the individual and tremendous costs for society [1]. In the Netherlands, the smoking prevalence rate is high, with 27.7% of the population above 15 years smoking [2]. A substantial part of Dutch adult smokers intend to quit smoking in the future [2,3]. Unfortunately, most quit attempts fail, and approximately three-quarters of unaided quitters resume smoking within three months [4].

Several intervention programs have been shown effective in increasing the chance of successful smoking cessation. However, only a minority of smokers makes use of such programs [5,6]. A possible explanation for this low rate may be that most programs rely on the smoker to take the initiative [7]. Proactive recruitment approaches to smoking cessation are scarce, even though such approaches may greatly enhance use of cessation support and, in turn, successful smoking cessation.

In the present study, smoking parents will be proactively recruited through their children's primary schools to participate in a randomized controlled trial evaluating the effectiveness of telephone counselling to aid smoking cessation. Telephone counselling has previously been shown to be effective in increasing smoking cessation rates in a meta-analytic review [8]. A recent Australian study utilized a proactive recruitment approach to increase smokers' use of telephone cessation support. In this study, 52% of identified smokers from a randomly called sample of 48,014 households agreed to participate in a randomized controlled trial to evaluate the effectiveness of telephone counselling in smoking cessation. Participants receiving telephone counselling were significantly more likely to report 7-day point prevalence abstinence at the 4-month (13.8% versus 9.6%) and 7-month assessment (14.3% versus 11%) compared to participants in the control condition [9]. As demonstrated in this study, proactive recruitment into telephone counselling seems an efficient way to increase use of cessation support and to enhance rates of smoking cessation in the general population.

In addition to the direct health benefits for smokers, smoking cessation of parents may have incremental effects for their children. Smoking behaviour of parents is an important risk factor for smoking initiation and smoking behaviour of children. A recent meta-analysis concluded that smoking behaviour of one parent significantly increases the child's risk to initiate smoking, and smoking behaviour of both parents adds to this risk [10]. As nicotine is severely addictive, experimentation with and uptake of smoking is hazardous behaviour. Prevention of children's exposure to factors that increase their risk of smoking initiation constitutes a significant task in tobacco control.

The effects of parental smoking on child smoking are likely to be mediated by children's smoking-related cognitions (e.g., attitudes towards smoking, normative beliefs about smoking, risk and benefit perceptions, tobacco refusal self-efficacy, intention to smoke). Previous research has shown that children of smoking parents are more likely to have more tolerant and positive attitudes towards smoking [11,12], more normative perceptions of smoking [13], and a stronger intention to smoke [12]. Smoking-related cognitions, in turn, have been established as prospective predictors of smoking initiation in adolescents [14,15].

Parental smoking cessation, however, has been shown to constitute an efficient way to decrease children's risk of smoking initiation [16]. The shorter the exposure to family models who smoke, the less likely it is that children will initiate smoking themselves [17]. The effect of parental smoking cessation has been shown to be mediated by their children's cognitions. In a recent study, 49% of the prospective relationship between parental smoking cessation and smoking behaviour of children was significantly mediated by negative attitudes toward smoking and tobacco refusal self-efficacy [18]. Presumably, telephone counseling to aid parental smoking cessation may have measureable effects in children of smoking parents as well.

### Aim and hypotheses

The primary aim of this study is to conduct a 2-arm randomized controlled trial to evaluate the effectiveness of proactive telephone counselling in increasing cessation rates among smoking parents. In addition, we will evaluate differences in smoking-related cognitions among children of parents in the telephone counselling and in the control condition. Three assessments among parents and children will take place (baseline, three months after start of the intervention, and twelve months after start of the intervention). Primarily, we expect higher smoking cessation rates among parents in the telephone counselling condition than in the control condition. Also, we expect children of parents in the telephone counselling condition to have more negative attitudes towards smoking, less normative perceptions of smoking, higher self-effectiveness to refrain from smoking, and a lower intention to start smoking than will children of parents in the control condition.

## Methods/Design

### Study Design

The present study is a 2-arm (telephone counselling versus control condition) randomized controlled trial with three assessments during a period of approximately one year. Participants will be 512 smoking parents and their 9-12 year old children. After giving informed consent and after completing the baseline assessment, 256 parents will be randomly assigned to the telephone counselling condition and 256 parents to the control condition. In the telephone counselling condition, parents will receive up to seven counsellor-initiated telephone calls and three supplementary brochures over a period of approximately three months. In the control condition, parents will receive a standard brochure on smoking cessation. Parent and child assessments will be identical across conditions and take place at baseline, three months after start of the intervention (post-measurement), and twelve months after start of the intervention (follow-up measurement). In both conditions, each parent-child couple will receive an incentive of 100 euro for their participation in all assessments.

### Participants

#### Recruitment

Smoking parents will be recruited through their children's primary schools. Primary school boards will be asked to distribute study invitation letters to all children aged 9-12 years and request that children give these letters to their parents. Study invitation letters include information about the study (e.g., purpose of study, length of the study, frequency of assessments, eligibility criteria). Parents will be able to register for the study by returning a form with their contact information in an enclosed envelope. Registration will also be possible via the study website, via e-mail, or via telephone.

#### Eligibility Criteria

Eligibility criteria are stated clearly in the study invitation letter. Inclusion criteria for the present study are: 1) being at least a weekly smoker, 2) being a parent/caretaker of a child in (Dutch) grade 6-8 (9-12 years old), 3) having the intention to quit smoking (currently or in the near future), and 4) giving informed consent. Upon registration, written informed consent of parents will be obtained. The ethics committee of the Faculty of Social Sciences at the Radboud University Nijmegen approved the study's protocol.

### Randomization

Assignment to a group will be performed by a member of the research group who is not involved in the present study. Participants will be stratified by gender, educational level, and smoking intensity (as reported by participants in the baseline questionnaire). If partners who live in the same household participate in the study, randomization will be carried out at household level to avoid contamination between conditions.

### Sample size calculation

Based on similar studies, we expected a 6% difference in 7-day point prevalence abstinence rates between the telephone counselling condition and the control condition at 12-months assessment (13% versus 7%, respectively). A statistical power of .80 was targeted. Hypotheses will be tested at a two-sided significance level of .05. The calculated sample size was corrected for participants who will be lost to attrition. Additionally, the sample size was corrected to allow for supplementary analyses of mediation and moderation.

### Study intervention

#### Theoretical basis of the intervention

Telephone counselling will be based on Motivational Interviewing (MI) and cognitive behavioural skill building. MI is considered a client-centered, directive method to enhance intrinsic motivation for behavioural change by exploring and resolving ambivalence [19]. MI's primary goal is to trigger a decision and enhance commitment to this decision, for example by eliciting and selectively reinforcing change talk. MI's empathic, non-confrontational style may be particularly helpful in addressing smokers' ambivalence and defensiveness and in providing a safe counselling environment for smoking parents. When parents express a desire to quit smoking, telephone counselling will shift to cognitive-behavioural skill building. Smokers will be encouraged to create a supportive environment for quitting (e.g., arrange for smoking substitutes, avoid exposure to smoking cues). The overall approach to skill building is a problem-solving one. Smokers are encouraged to identify key barriers to quitting and to remaining quit (e.g., stress, urges and cravings, exposure to smoking cues, dysfunctional cognitions), to identify practical solutions, and to implement and evaluate these solutions. Cognitive-behavioural skill building will also incorporate relapse prevention strategies (e.g., anticipation of difficult situations/lapse to smoking). During telephone counseling, motivation to quit and self-efficacy to quit will be continuously monitored by counsellors. Counsellors will alternate MI and cognitive-behavioural skill building according to the participant's current need for motivation enhancement or skill enhancement.

#### Telephone counselling condition

In the telephone counselling condition, parents receive proactive telephone counselling based on MI and cognitive-behavioural skill building. Each participant receives up to seven counsellor-initiated phone calls across a period of approximately three months, respectively one 30-minute intake session and up to six additional 10-minute sessions. Telephone counselling will be conducted by professionals of STIVORO (Dutch expert centre for tobacco control). All counsellors are trained and experienced in the delivery of telephone counselling to support smoking cessation. Two different call schedules will be applied to participants who are willing to set a quit date and participants who are not willing to set a quit date.

##### Participants who are willing to set a quit date

Participants who are willing to set a quit date are offered 1-2 preparatory phone calls before undertaking a quit attempt. During the first phone call, participants are encouraged to set a quit date within 10-12 days. In the following, participants are offered up to six phone calls to support maintenance of smoking cessation. The phone calls focus on the following topics: reasons for smoking and reasons for quitting, nicotine dependence and nicotine withdrawal, craving, coping with difficult situations, weight gain and irritability, and relapse prevention. The first phone call (intake call) will take place approximately 10-12 days before the quit date; the second phone call will take place approximately three days after quit date; the third phone call approximately seven days after quit date; the fourth phone call approximately two weeks after quit date; the fifth phone call approximately four weeks after quit date; the sixth phone call approximately eight weeks after quit date; and the seventh phone call approximately twelve weeks after quit date.

##### Participants who are not willing to set a quit a date

Participants who are not willing to set a quit date will receive three phone calls. These phone calls are intended to increase the participant's motivation for smoking cessation by use of Motivational Interviewing. During these calls, counsellors aim to explore the participant's reasons for smoking and for quitting, to resolve ambivalence, and to enhance the participant's intrinsic motivation for behavioural change. Participants will receive the second phone call approximately three weeks after the first phone call (intake call). Approximately four weeks later the third phone will be made.

##### Supplementary brochures

All participants in the telephone counselling condition will receive three supplementary brochures on smoking cessation. All brochures are 4-page, colour-printed, A4-sized booklets which are designed specifically for the present study. The brochures have the following themes: 1) Deciding and preparing, 2) Undertaking a quit attempt, 3) Maintenance of smoking cessation. Each brochure includes additional information about smoking and smoking cessation, tips and exercises, and motivation or self-efficacy enhancing messages. Additionally, each brochure contains information which is relevant to parents (e.g., information about effects of second-hand smoke exposure for children). Participants will receive the first brochure immediately after start of the telephone counselling, the second brochure approximately 2-3 weeks after start of the telephone counselling, and the last brochure approximately 5-6 weeks after start of the telephone counseling.

#### Control condition

Participants in the control condition will receive a standard brochure (by STIVORO) on smoking cessation (Stoppen met roken: Willen en kunnen [Quitting smoking: Wanting to quit and being able to quit]). The brochure is a 40-page, colour-printed booklet (size: 12 × 16 centimeters). The brochure will be sent to participants randomized to the control condition within two weeks after baseline assessment. The brochure is divided into 5 parts: information about smoking and smoking cessation, reasons for quitting, tips and exercises, and maintenance of smoking cessation. At the end of the study, telephone counselling will be offered to all participants in the control condition.

### Data collection

**Figure 1:**
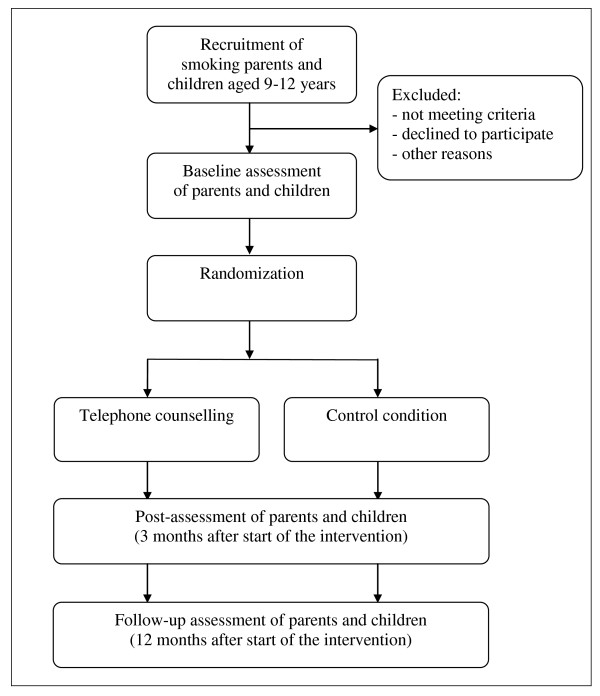
**Study design**.

An overview of the study design is presented in Figure 1. The baseline measurement will take place between January and July 2011. It is expected that the majority of the questionnaires will be administered digitally (the rest will be administered via mail). The post-measurement will take place approximately three months after start of the intervention. The follow-up assessment follows approximately twelve months after start of the intervention. At all three assessments, questionnaires will be filled in by both the parent and the child. Procedures will be identical across assessments.

### Outcomes

In the proposed study, telephone counselling aims to increase cessation rates among smoking parents. The primary outcome measures will be: 1) sustained abstinence between post-measurement and follow-up measurement, 2) 7-day point prevalence abstinence at post-measurement (three months post-intervention) and follow-up measurement (twelve months post-intervention), and 3) 24-hours point prevalence abstinence at post-measurement and follow-up measurement. Additionally, biochemical validation of self-reported smoking cessation will be reported for a random sample (30%) of all study participants who report 7-day point prevalence at follow-up assessment, thereby allowing to estimate the occurrence of over-reporting of abstinence. Secondary outcome measures will include: a 50% reduction in the number of cigarettes smoked per day, occurrence of abstinence of at least 24 hours at some point during the study, implementation of smoking restrictions at home, increase in motivation to quit, use of and adherence to nicotine replacement therapy, number and duration of quit attempts, and change in smoking-related cognitions (e.g., attitudes towards smoking, self-efficacy, social norms). In addition, secondary outcomes will include smoking-related cognitions of children (e.g., attitudes towards smoking, self-efficacy, social norms, intention to smoke) and smoking behaviour of children.

### Statistical analyses

Analyses will be conducted to check whether the randomization has resulted in an equal baseline distribution of relevant participant characteristics across both conditions. In case of group differences at baseline, confounding variables will be included in subsequent analyses. To evaluate smoking cessation rates across groups, we will use logistic regression models. Effect sizes as well as confidence intervals will be reported. To evaluate smoking-related cognitions across groups (in both parents and children), analyses-of-variance and regression analyses will be used. Mediation and moderation will be tested in Mplus. In accordance with the intent-to-treat principle, all participants randomized to a condition will be included in analyses testing of the study hypotheses. In addition, a complete-case analysis will also be conducted, that is, only participants with outcome data on all assessments will be included in the analysis.

## Discussion

The present study protocol presents the design of a randomized controlled trial evaluating the effectiveness of proactive telephone counselling in smoking parents. The purpose of telephone counselling is to increase smoking cessation rates among parents. We hypothesize that cessation rates will be higher in the telephone counselling condition compared to the control condition, both at three-months post-measurement as well as twelve-months follow-up measurement. Additionally, we hypothesize that children of parents receiving telephone counselling will have more negative attitudes towards smoking, perceive stronger social norms against smoking, have higher self-efficacy to refrain from smoking, and have a lower intention to start smoking than will children of parents in the control condition.

### Strengths and limitations

Strengths of the study include a 12-month follow-up assessment, which meets smoking cessation research recommendations [20]. A limitation of the study is that smoking cessation will be assessed by self-report. However, the present study counteracts reporting biases by informing participants that a random sample of participants will be asked for biochemical validation of self-reported smoking cessation. Biochemical validation of self-reported smoking cessation will be reported for a subsample (30%) of all study participants who report 7-day point prevalence at follow-up assessment, thereby allowing to correct for over-reporting of abstinence. Another potential limitation is that the impact of the intervention on children's cognitions about smoking may be limited by the degree to which their parents quit smoking.

### Implications for practice

Results of the present study can be of help in adapting telephone counselling and in tailoring telephone counselling to the needs of particular subgroups. If the intervention is found effective, it can be advertised through schools to reach the population of smoking parents. If children are found to benefit from this intervention, proactive recruitment of smoking parents into telephone counselling may be incorporated in national prevention campaigns, such as the "Healthy School and Drugs" program [21], which has already been implemented in numerous schools, institutions, and treatment facilities.

## Conclusions

The proposed study will evaluate the effectiveness of proactive telephone counselling to aid smoking cessation among smoking parents. Additionally, it will evaluate whether children of smoking parents receiving telephone counselling have less favorable cognitions about smoking than do children of parents in the control condition. The results of this study will provide insight into parent characteristics and intervention characteristics associated with successful smoking cessation. In addition, the proposed study will provide insight into the intergenerational transmission of smoking-related cognitions as well as the associates and antecedents of favorable smoking-related cognitions in preadolescents.

## Competing interests

The authors declare that they have no competing interests.

## Authors' contributions

KS is responsible for the data collection, data analysis, and report of study results. RO, MK, JB, and RE are supervisors and grant applicators. All authors read and approved the final manuscript.

## Pre-publication history

The pre-publication history for this paper can be accessed here:

http://www.biomedcentral.com/1471-2458/11/732/prepub

## References

[B1] World Health Organization: WHO report on the global tobacco epidemic 2008 Geneva

[B2] STIVORO: Smoking, the hard facts: Adults 2009 2009 Den Haag [Roken, de harde feiten: Volwassenen 2009]

[B3] Willemsen MC The new EU cigarette health warnings benefit smokers who want to quit the habit: Results from the dutch continuous survey of smoking habits Eur J Public Health 2005 15 398 392 10.1093/eurpub/cki061 15975953

[B4] Powell J Dawkins L West R Powell J Pickering A Relapse to smoking during unaided cessation: Clinical, cognitive and motivational predictors Psychopharmacology 2010 212 537 549 10.1007/s00213-010-1975-8 20703450

[B5] Hughes JR Marcy TW Nauds S Interest in treatments to stop smoking J Subst Abuse Treat 2009 36 18 24 10.1016/j.jsat.2008.04.002 18550319 PMC2635950

[B6] Swartz Woods S Haskins AE Increasing reach of quitline services in a US state with comprehensive tobacco treatment Tob Control 2007 16 Suppl.1 33 36 10.1136/tc.2007.019935 PMC2598522 18048629

[B7] Friend K Levy DT Smoking treatment interventions and policies to promote their use: A critical review Nic and Tob Res 2001 3 299 310 10.1080/14622200110072165 11694197

[B8] Stead LF Perera R Lancaster T telephone counseling for smoking cessation Cochrane Database of Systematic Reviews 2006 Issue 3 Art. No.: CD002850 10.1002/14651858.CD002850.pub2 16855992

[B9] Tzelepis F Paul CL Wiggers J Walsh RA Knight J Dunan SL Lecathelinais C Girgis A Daly J A randomised controlled trial of proactive telephone counselling on cold-called smokers' cessation rates Tob Control in press 10.1136/tc.2010.035956 PMC3003878 21030529

[B10] Leonardi-Bee J Jere ML Britton J Exposure to parental and sibling smoking and the risk of smoking uptake in childhood and adolescence: A systematic review and meta-analysis Thorax in press 10.1136/thx.2010.153379 21325144

[B11] Brook U Mendelberg A Galili A Priel I Bujanover Y Knowledge and attitudes of children towards cigarette smoking and its damage Patient Educ Couns 1999 37 49 53 10.1016/S0738-3991(98)00101-3 10640119

[B12] Porcellato L Dugdill L Springett J Sanderson FH Primary schoolchildren's perceptions of smoking: Implications for health education Health Edu Res 1999 14 71 83 10.1093/her/14.1.71 10537949

[B13] Otten R Engels RCME Prinstein MJ A prospective study of perception in adolescent smoking J Adolesc Health 2009 44 478 484 10.1016/j.jadohealth.2008.09.004 19380096 PMC4624098

[B14] Carvajal SC Wiatrek DE Evans RI Knee CR Nash SG Psychosocial determinants of the onset and escalation of smoking: Cross-sectional and prospective findings in multiethnic middle school sample J Adolesc Health 2000 27 255 265 10.1016/S1054-139X(00)00124-5 11008088

[B15] Song AV Morrel HE Cornell JL Ramos ME Biehl M Kropp RY Halpern-Felsher BL Perceptions of smoking-related risks and benefits as predictors of adolescent smoking initiation Am J Public Health 2009 99 487 492 10.2105/AJPH.2009.179010 19106420 PMC2661432

[B16] Bricker JB Leroux BG Peterson AV Jr Kealey KA Sarason IG Andersen MR Marek PM Nine-year prospectve relationship between parental smoking cessation and child's daily smoking Addiction 2006 98 585 593 10.1046/j.1360-0443.2003.00343.x 12751972

[B17] Gilman SE Rende R Boergers J Abrams DB Buka SL Clark MA Colby SM Hitsman B Kazura AN Lipsitt LP Lloyd-Richardson EE Rogers ML Stanton CA Stroud LR Niaura RS Parental smoking and adolescent smoking initiation: An intergenerational perspective on tobacco control Pediatrics 2009 123 274 281 10.1542/peds.2008-2251 PMC2632764 19171580

[B18] Wyszynski CM Bricker JB Comstock BA Parental smoking cessation and child daily smoking: A 9-year longitudinal study of mediation by child cognitions about smoking Health Psychol 2011 30 171 176 21401251 10.1037/a0022024 PMC3064434

[B19] Miller WR Rollnick S Motivational Interviewing: Preparing people to change 2002 2 New York: Guilford Press

[B20] Hughes JR Keely JP Niaura RS Ossip-Klein DJ Richmond RL Swan GE Measures of abstinence in clinical trials: Issues and recommendations Nic Tob Res 2003 5 13 25 12745503

[B21] Malmberg M Overbeek G Kleinjan M Vermulst A Monshouwer K Lammers J Vollebergh WAM Engels RCME Effectiveness of the universal prevention program ‘Healthy School and Drugs': Study protocol of a randomized clustered trial BMC Public Health 2010 10 541 10.1186/1471-2458-10-541 20825669 PMC2941689

